# Value of synaptic proteins as biomarkers in amyotrophic lateral sclerosis

**DOI:** 10.1093/braincomms/fcag247

**Published:** 2026-06-25

**Authors:** Frederik Hobin, Shreyasee Das, Charlotte Lambrechts, Charlotte De Rocker, Jonas Dubin, Fouke Ombelet, Joke De Vocht, Nikita Lamaire, Eugeen Vanmechelen, Koen Poesen, Philip Van Damme

**Affiliations:** Department of Neurology, University Hospitals Leuven, 3000 Leuven, Belgium; Department of Neurosciences, Laboratory of Neurobiology, Leuven Brain Institute (LBI), KU Leuven, 3000 Leuven, Belgium; Department of Biomarker Development, Fujirebio Europe NV, 9052 Ghent, Belgium; Neurochemistry Laboratory, Department of Clinical Chemistry, Amsterdam Neuroscience, Program Neurodegeneration, Amsterdam UMC, Vrije Universiteit Amsterdam, 1011 Amsterdam, The Netherlands; Department of Biomarker Development, Fujirebio Europe NV, 9052 Ghent, Belgium; Department of Biomarker Development, Fujirebio Europe NV, 9052 Ghent, Belgium; Laboratory of Molecular Neurobiomarker Research, Department of Neurosciences, Leuven Brain Institute (LBI), KU Leuven, 3000 Leuven, Belgium; Laboratory Medicine, UZ Leuven- University Hospitals Leuven, 3000 Leuven, Belgium; Department of Neurology, University Hospitals Leuven, 3000 Leuven, Belgium; Department of Neurology, University Hospitals Leuven, 3000 Leuven, Belgium; Department of Neurosciences, Laboratory of Neurobiology, Leuven Brain Institute (LBI), KU Leuven, 3000 Leuven, Belgium; Department of Neurology, University Hospitals Leuven, 3000 Leuven, Belgium; Department of Biomarker Development, Fujirebio Europe NV, 9052 Ghent, Belgium; Laboratory of Molecular Neurobiomarker Research, Department of Neurosciences, Leuven Brain Institute (LBI), KU Leuven, 3000 Leuven, Belgium; Laboratory Medicine, UZ Leuven- University Hospitals Leuven, 3000 Leuven, Belgium; Department of Neurology, University Hospitals Leuven, 3000 Leuven, Belgium; Department of Neurosciences, Laboratory of Neurobiology, Leuven Brain Institute (LBI), KU Leuven, 3000 Leuven, Belgium

**Keywords:** biomarker, amyotrophic lateral sclerosis, synapse, cerebrospinal fluid, prognosis

## Abstract

Amyotrophic lateral sclerosis is a heterogeneous and rapidly progressing neurodegenerative disorder with limited treatment options. Therefore, there is a critical need for biomarkers that capture the diverse pathophysiological mechanisms underlying disease onset and progression. Emerging evidence suggests that synaptic dysfunction is an early disease mechanism in amyotrophic lateral sclerosis. Using homebrew immunoassays, we explored a panel of pre- and post-synaptic proteins in cerebrospinal fluid of patients with amyotrophic lateral sclerosis (*N* = 57) and controls (*N* = 36). The potential value as a biomarker was explored by correlating cerebrospinal fluid levels with clinical parameters and established biomarkers for amyotrophic lateral sclerosis. Higher levels of Neurogranin (NRGN) (*P* = 0.003) and Vesicle-associated membrane protein 2 (VAMP2) (*P* = 0.014) were observed in patients with amyotrophic lateral sclerosis compared with controls. VAMP2, Synaptosome-associated protein 25 kDa (SNAP25) and β-synuclein (SNCB) correlated with individual relative disease stage, but none of the biomarkers correlated with disease progression rate. High levels of SNAP25 predicted worse survival in a univariate and stepwise multivariable analysis, but significance did not persist upon including Neurofilament light chain (NfL) levels. Synaptic proteins did not correlate with cerebrospinal fluid levels of neurofilaments or biomarkers of neuroinflammation, suggesting that they reflect different pathological mechanisms in amyotrophic lateral sclerosis. Our findings warrant further investigation to determine whether increased cerebrospinal fluid levels of synaptic proteins reflect synaptic breakdown or active release of synaptic proteins. This will help elucidate how synaptic dysfunction or damage contributes to elevated levels of synaptic markers in amyotrophic lateral sclerosis, and its underlying value as biomarker.

## Teaser

In this study, Hobin *et al.* identify synaptic proteins as novel biomarkers in amyotrophic lateral sclerosis (ALS), reporting increased cerebrospinal fluid levels of some synaptic proteins in ALS patients. Hobin *et al.* report that certain synaptic proteins correlate with relative disease stage or can predict survival within ALS patients.

## Graphical Abstract

**Graphical Abstract:**
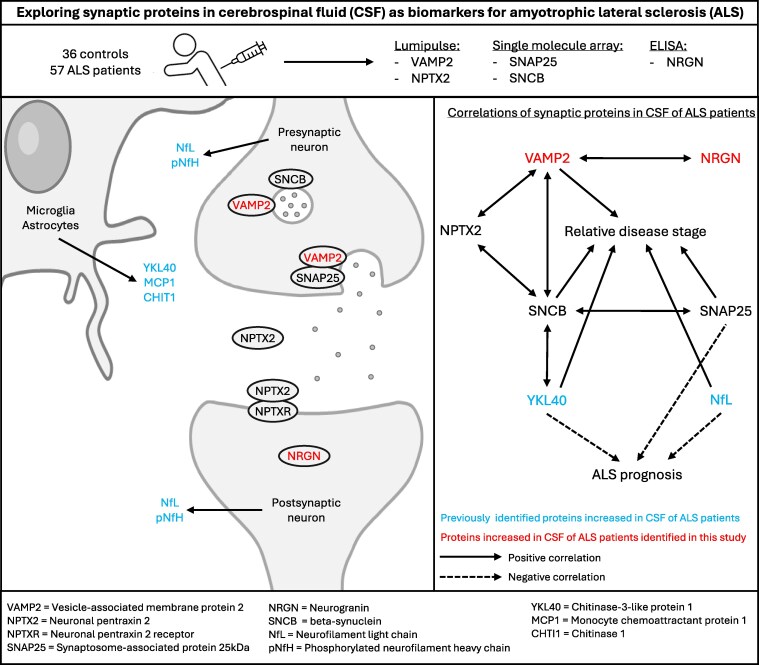

## Introduction

Amyotrophic lateral sclerosis (ALS) is a progressive motor neuron disease, affecting both upper and lower motor neurons in the motor cortex, brain stem and spinal cord. Since there is no cure available, it results in progressive muscle weakness and wasting, with respiratory failure on average 2–5 years after symptom onset.^1^ In about 15–20% of cases, a genetic mutation is identified as the underlying cause of the disease.^2,3^ The most commonly mutated genes include *Chromosome 9 open reading frame 72* (*C9orf72*), *Superoxide dismutase 1* (*SOD1*), *TAR DNA binding protein 43 kDa* (*TARDBP*), *Fused in sarcoma* (*FUS*) and *TANK-binding kinase 1* (*TBK1*). However, in the remaining 80–85% of cases, the cause remains unknown. Many disease mechanisms have been implicated in the loss of motor neurons in ALS, including alterations in RNA metabolism, protein degradation machinery, axonal and synaptic function and neuroinflammation.^1^

Besides neuro-axonal damage and neuroinflammation, synaptic dysfunction and loss has been observed in multiple ALS models as pathological hallmarks contributing to motor neuron degeneration.^4,5^ It is hypothesized that synaptic dysfunction is an early event in ALS that aggravates over time, and precedes motor neuron loss.^4^ Indeed, presynaptic deterioration is present in *C9orf72* cortical neurons harbouring *C9orf72* repeat expansions and motor neurons in mutant *SOD1 G93A* mice.^6,7^ Interestingly, mutant *TDP-43* causes changes in spine density associated with changes in the excitatory/inhibitory (E/I) balance, even at presymptomatic stages.^8^ Moreover, recent evidence suggests that spinal inhibitory input is lost before loss of motor neurons in *SOD1 G93A* mice.^9^ Genetic evidence for the importance of synaptic dysfunction in ALS comes from the discovery of an intronic SNP in *UNC13A* which is seen as a risk factor in ALS, associated with cortical atrophy, cognitive impairment and shorter survival.^10,11^ Importantly, reduced nuclear function of TDP-43 has been shown to induce mis-splicing of *UNC13A*, resulting in reduced *UNC13A* levels by inducing an out-of-frame cryptic exon prone to non-sense mediated mRNA decay.^12,13^ In addition, TDP-43 pathology induces cryptic exons in a broad range of proteins involved in synaptic function.^14^ Moreover, TDP-43 has been shown to bind and control levels of Neuronal Pentraxin 2 (NPTX2), a regulator of excitatory synapse formation, which accumulates and induces neurotoxicity upon TDP-43 pathology.^15^ Altogether, synaptic dysfunction may be an important disease mechanism in ALS and that synaptic dysfunction may contribute to disease progression in ALS.

Cerebrospinal fluid (CSF) levels of neurofilaments [i.e. neurofilament light chain (NfL) and phosphorylated neurofilament heavy chain (pNfH)] are well-established biomarkers of neuronal/axonal injury in ALS as they rise early in the disease, predict survival at onset and remain relatively stable during the course of the disease.^16-18^ NfL and pNfH levels have become an important read-out of treatment effects in ALS trials.^19,20^ In addition, previous research demonstrated that CSF levels of monocyte chemoattractant protein 1 (MCP-1), chitinase-like protein 3 (YKL-40) and chitinase-1 (CHIT-1) are increased in ALS and contribute to survival, reflecting pathological neuroinflammation.^21,22^ However, biomarkers that evolve with disease stage are scarce. Moreover, a panel of fluid biomarkers that accurately captures the full spectrum of disease mechanisms underlying motor neuron degeneration is largely missing. We hypothesize, given the observed synaptopathy present early in the disease trajectory of ALS that synaptic proteins could be of importance for ALS. Here, our aim was to explore the biomarker potential of synaptic proteins in ALS. We therefore measured a set of synaptic proteins in CSF of ALS patients and tested their prognostic value and correlations with clinical parameters.

## Materials and methods

### Biofluid sample collection

Upon obtaining informed consent, CSF was collected within the University Hospitals Leuven (UZ Leuven) from controls and sporadic ALS patients through lumbar puncture (LP) at the diagnostic stage between 2010 and 2023. CSF samples were collected and processed in the laboratory medicine department of UZ Leuven. Upon centrifugation, samples were aliquoted and stored in −80°C for further biomarker measurements. Within ALS patients, mean time between symptom onset and CSF measurement was 13.27 ± 8.30 months. The control group consisted of 22 people with subjective cognitive impairment, 9 people with migraine, 2 people with subjective headache, 1 person with a tremor, 1 person with primary cough headache and 1 person with gait impairment of non-neurological origin. All controls had normal neurological investigations. This study is approved by the ethical committee of the University Hospitals Leuven (UZ Leuven).

### Clinical parameters

Clinical data were collected from medical records at UZ Leuven. Disease duration was measured as months between symptom onset and CSF measurement. ALS-FRS-R slope was determined as the number of points lost per month, by dividing the difference in scores between the first and last available score by the time between the measurements in months. Slow, normal and fast progression was measured by an ALS-FRS-R slope of <0.3, 0.3><0.9, or >0.9, respectively.^23^ Disease progression rate was calculated as (48—ALS-FRS-R score at diagnosis) divided by diagnostic delay in months. To investigate correlations with electrophysiological measures of ALS pathology, we used outcome measures of diagnostic electromyography (EMG) testing that indicate lower motor neuron involvement. The number of regions affected based on EMG is defined by the number of Revised El Escorial score. We calculated a combined compound muscle action potential (CMAP) score by summing the CMAP values derived from stimulation of the median nerve (innervating the abductor pollucis brevis) and the peroneal nerve (innervating the extensor digiti minimi).

### CSF biomarker measurements

Synaptic proteins measured in CSF are Neurogranin (NRGN), Vesicle-associated membrane protein 2/Synaptobrevin-2 (VAMP2), β-synuclein (SNCB), Neuronal pentraxin 2 (NPTX2) and Synaptosome-associated protein 25 kDa (SNAP25). Synaptic proteins were either measured with assays developed by Fujirebio Europe NV (formerly ADx NeuroSciences NV) (SNAP25, VAMP2, SNCB, NPTX2) or using commercially available immunoassays (NRGN).

SNAP25 was measured using a concept homebrew immunoassay developed by Fujirebio Europe NV (formerly ADx NeuroSciences NV) on the Quanterix SIMOA HD-X platform. The qualification results and specifications of this assay have been published elsewhere.^24^ In short, a mouse monoclonal antibody, ADx404, targeting the N-terminal end of human SNAP25 was used as capture antibody. RD-086, recognizing an internal epitope in the N-terminus was used as detector. A synthetic peptide was used to convert signals to concentrations.

The SNCB assay is also a homebrew immunoassay on the SIMOA HD-X platform with two newly generated, highly SNCB-specific monoclonal antibodies which have not yet been described. ADx415, a rabbit monoclonal antibody is used to capture the antigen, while ADx416 is used as detector.^25^ The assay is calibrated with a recombinant SNCB protein.

VAMP2 and NPTX2 were measured using Xplorer assays (ADxPLORER). The VAMP2 Xplorer v1 kit uses a monoclonal antibody, ADx413, with an internal epitope as capture and a new N-terminal specific monoclonal antibody, ADx414, as detector. The assay is calibrated using a synthetic peptide. The NPTX2 Xplorer kit uses ADx410, an IgG2a, as capture and ADx409, an IgG1, as detector (antibodies have been previously described).^26^ The assay is calibrated with a purified recombinant NPTX2 protein.

Neurogranin CSF levels have been determined using a commercially available neurogranin kit according to the manufacturer’s instructions (EQ 6551-9601-L, Euroimmun AG, Lübeck, Germany).^27^

In the SNCB assay, one patient measurement failed and in the VAMP2 assay, one patient and one control measurement failed, resulting in a slight difference in sample size.

### Statistics

Statistical data and figures were analysed and created using IBM SPSS Statistics (Version 30.0.0.0), Graphpad Prism (Version 10.6.1) or RStudio (RStudio: Integrated Development for R. Version 2025.09.2 + 418). Normality of data was assessed using Shapiro-Wilk testing. Significance of numeric data (Age at LP) was assessed using a T test. Significance of categorical data (sex) was assessed using a Chi^2^ test. To account for age-related influences in results, CSF protein measurements were compared with non-parametric Quade ANCOVA, using age as covariate. Correlation analyses of normally or non-normally distributed data were performed by a Pearson or Spearman correlation, respectively. Lasso regression was performed on normalized biomarker data using *z*-scores. Using the *glasso* R package, we computed a Gaussian graphical model using graphical lasso with a chosen tuning of 0.05 based on the extended Bayesian information criterium (EBIC). Stepwise linear regression was performed using synaptic proteins as dichotomous variables (i.e. above and below median CSF levels) to investigate relative contributions of proteins to survival. Kaplan-Meier analyses were performed for survival analyses of biomarker levels as dichotomous variable. Significance of Kaplan-Meier analyses was assessed using Log rank test (Mantel-Cox). Normalized values of biomarker levels, centred values of age and scaled values of diagnostic delay were used as continuous variables for Cox regression analyses. Significance of multivariate survival analyses was assessed using Cox regression analyses, and significance of each covariate was calculated by Chi^2^ testing with respective hazard ratios (HR) with 95% confidence intervals (CI 95%). In all analyses, statistical significance was attained at a *P*-value < 0.05. Additionally, Bonferroni correction was added in all analyses for multiple correction testing and adjusted *P*-values are displayed.

## Results

### Some synaptic proteins are increased in CSF of ALS patients, but do not correlate with parameters of ALS progression

We explored a set of synaptic proteins and their potential as biomarkers in ALS patients and controls. The demographics of the study population are shown in Table 1. Age at LP was higher in the ALS group. The synaptic proteins included proteins with presynaptic (SNAP25, VAMP2, SNCB) and post-synaptic (NPTX2, NRGN) functions that were tested in CSF of sporadic ALS cases and controls. Analytical performance of all immunoassays is shown in Table 2.

**Table 1:** Demographics of the study population

	CTRL (*N* = 36)	ALS (*N* = 57)	*P*-value
Age at LP (mean ± SD)	58.60 ± 11.28	64.46 ± 11.25	***0***.***018***
Male sex (%)	18 (51.43%)	34 (59.70%)	*0*.*32*
Spinal onset (%)	/	42 (73.68%)	*/*
Disease duration (mean ± SD)	/	13.27 ± 8.30	*/*
CSF levels of synaptic protein (mean concentration in pg/mL)			
NRGN	140.4	198.1	***0***.***003***
VAMP2	1435.1	1725.1	***0***.***014***
SNAP25	193.7	168.0	*0*.*061*
NPTX2	1624.1	1404.0	*0*.*27*
SNCB	640.7	740.3	*0*.*41*

T test was used to compare age at lumbar puncture (LP). Chi^2^ test was used to compare sex. CSF protein levels were compared with Quade ANCOVA test using age as covariate. For CSF levels: NRGN: N ALS = 57; N CTRL = 36; VAMP2: N ALS = 56; N CTRL = 35; SNAP25: N ALS = 57; N CTRL = 36; NPTX2: N ALS = 57; N CTRL = 36; SNCB: N ALS = 56; N CTRL = 36. Bonferroni correction: *P* = 0.01. SD, standard deviation. Significant *P*-values are displayed in bold. CTRL, controls.

**Table 2:** Analytical parameters for all immunoassays

Performance					
	NRGN	SNAP25	SNCB	VAMP2	NPTX2
	ELISA	Simoa	Simoa	Lumipulse	Lumipulse
Parameter					
Measured clinical range	74.08–938.46	0.34–7.20	7.42–51.24	159.79–1134.30	51.70–411.14
Dilution factor CSF	1	120	30	4	10
Clinical range with dilution factor	74.08–938.46	40.80–863.64	222.49–1537.13	639.15–4537.20	517.04–4111.35
Variation in reproducibility	7%	2.3–3.7%	4.6%	4.3%	8.2%
Dilutional linearity	Up to 10	85–125%	Not done	Up to 5000	Up to 20
Spike-recovery	Not done	79–106%	77–102%	98–120%	100–110%
Stability	Not done	Not done	3.12%	4.29%	Not done

Stability is defined as inter-run precision of quality control samples. Ranges are defined in pg/mL.

**Figure 1:**
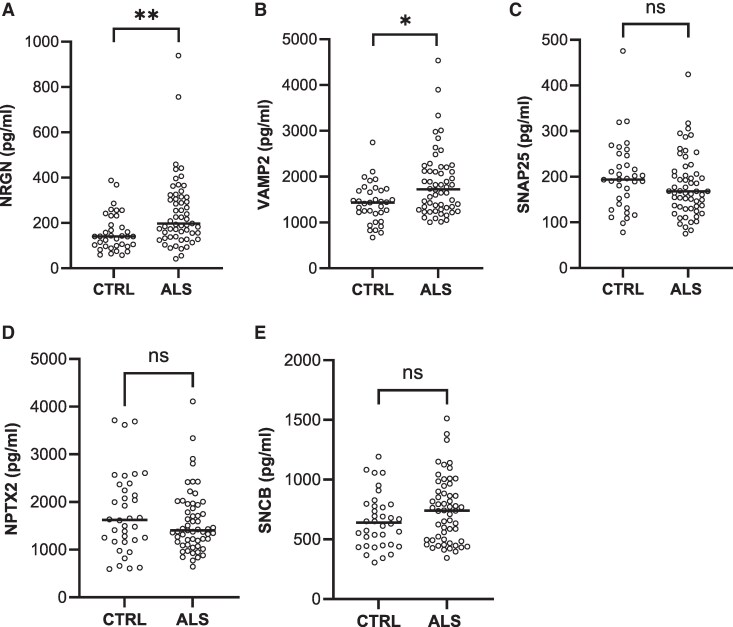
**CSF levels of NRGN and VAMP2 are increased in ALS patients.** (**A**) NRGN CSF measurements: ANCOVA *P* = 0.003; N ALS = 57; N CTRL = 36. (**B**) VAMP2 CSF measurements: ANCOVA *P* = 0.014; N ALS = 56; N CTRL = 35. (**C**)SNAP25 CSF measurements: ANCOVA *P* = 0.061; N ALS = 57; N CTRL = 36. (**D**) NPTX2 CSF measurements: ANCOVA *P* = 0.27; N ALS = 57; N CTRL = 36. (**E**) SNCB CSF measurements: ANCOVA *P* = 0.41; N ALS = 56; N CTRL = 36. Data points represent biomarker levels from individual ALS patients or controls. *P < 0.05; **P < 0.01. NS = not significant. Bonferroni correction: *P* = 0.01. CTRL = controls.

CSF levels of VAMP2 and NRGN were significantly increased in ALS patients compared with controls. (Figure 1), unlike CSF levels of SNAP25, SNCB and NPTX2. Next, we analysed whether CSF levels of synaptic proteins correlated with clinical parameters of disease progression. We did not find any correlations between the synaptic protein levels in CSF and clinical parameters of disease progression (Supplementary Table 1). Moreover, none of the synaptic proteins in CSF correlated with age or weight in controls (Supplementary Table 2).

Based on our hypothesis that the increase of synaptic proteins depends on the stage of the disease, we calculated a value for relative disease stage formulated by:


$$\frac{\left(\mathrm{Time}\;\mathrm{in}\;\mathrm{months}\;\mathrm{between}\;\mathrm{symptom}\;\mathrm{onset}\;\mathrm{and}\;\mathrm{lumbar}\;\mathrm{puncture}\right)}{\left(\mathrm{Time}\;\mathrm{in}\;\mathrm{months}\;\mathrm{between}\;\mathrm{symptom}\;\mathrm{onset}\;\mathrm{and}\;\mathrm{death}\right)}$$

**Figure 2:**
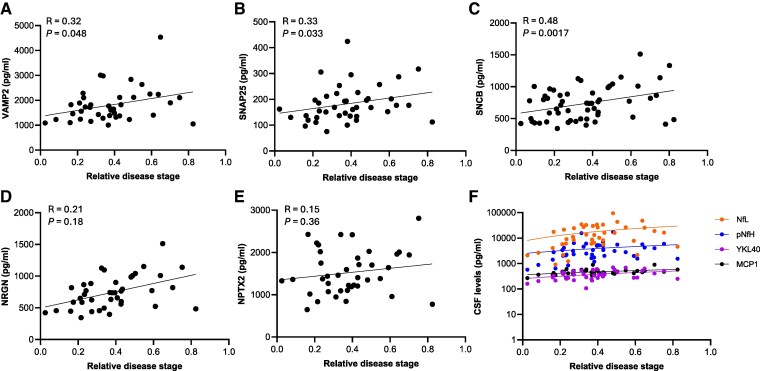
**Synaptic proteins in CSF correlate with relative disease stage.** CSF levels of SNAP25, SNCB and VAMP2 positively correlate with the relative disease stage at time of sampling. Spearman correlations were performed for all biomarkers. (**A–E**) Spearman R and *P*-values are shown on the figure. Bonferroni correction: *P* = 0.01. *N* = 41 for SNAP25, NRGN and NPTX2. *N* = 40 for VAMP2 and SNCB. (**F**) NfL: Spearman R = 0.39; *P* = 0.013. pNfH: Spearman R = 0.36; *P* = 0.022; YKL-40: Spearman R = 0.41; *P* = 0.0078. MCP-1: Spearman R = 0.35; *P* = 0.026. *N* = 41 for all biomarkers in panel (F). Data points represent biomarker levels from individual ALS patients.

This measure can be used as a personalized indicator of relative disease stage (relative disease duration) at the time of the biomarker measurement for each patient. Interestingly, we observed a positive correlation between this relative measure and levels of VAMP2, SNAP25 and SNCB, suggesting that higher CSF levels reflect relatively further disease progression at the time of the biomarker measurement.(Figure 2A–E) This is in line with what is seen for CSF levels of neurofilaments and inflammatory proteins, both established ALS biomarkers (Fig. 2F).

We also compared the levels of synaptic biomarkers to the number of regions with lower motor neuron involvement based on EMG (according to the Revised El Escorial criteria) and King’s stage. CSF levels did not differ between patients with a different number of regions affected based on EMG or between patients in different King’s stages (Supplementary Figs 1 and 2).

### Prognostic value of synaptic proteins in ALS

We first compared the levels of synaptic proteins between slow, intermediate, and fast disease progressors, as defined by the monthly ALS-FRS-R decline. Although an increasing stepwise trend is seen in all synaptic markers between slow, intermediate and fast progressors, this did not reach significance. Only CSF NRGN levels tended towards significantly higher levels in fast progressing patients (*P* = 0.08) (Supplementary Fig. 3).

**Figure 3:**
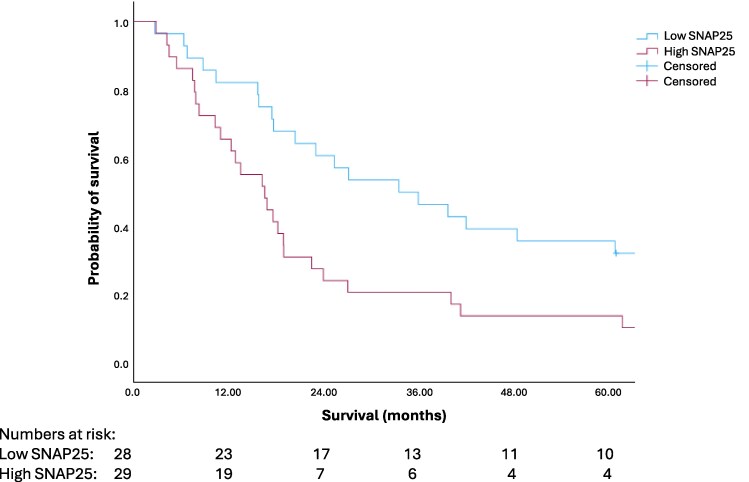
**Survival analysis of low and high CSF SNAP25 levels.** Kaplan-Meier analysis of CSF SNAP25 levels as dichotomous variable. Log rank *P*-value = 0.011. *N* = 57. Censored values are indicated between brackets.

To further establish the prognostic value of these synaptic proteins, we performed survival analyses. To investigate the relative contribution of each synaptic protein to survival, a stepwise linear regression analysis was performed on dichotomous levels of all proteins (i.e. above and below median) (Table 3). This resulted in high CSF SNAP25 levels as strongest contributor to survival. Subsequently, Kaplan-Meier analyses were performed on every synaptic biomarker, divided into high and low levels based on median CSF levels. These analyses confirmed that high CSF SNAP25, although not significantly increased compared with controls, was the only significant contributor to survival (*P* = 0.011) (Figure 3; Supplementary Fig. 4).

**Table 3:** Stepwise regression analysis of synaptic proteins contributing to survival

	Standardized coefficients beta	95% CI	T-value	*P*-value
SNAP25	−0.418	[−50.69; −7.63]	−2.722	*0*.*009*
NRGN	−0.183	[−33.44; 7.90]	−1.241	*0*.*220*
VAMP2	0.276	[−15.62; 54.18]	1.110	*0*.*272*
NPTX2	−0.144	[−42.82; 22.75]	−0.615	*0*.*451*
SNCB	0.11	[−24.73; 40.07]	0.476	*0*.*636*

Synaptic proteins are included as dichotomous variable (i.e. above and below median levels). CI, confidence interval.

Next, multivariate Cox regressions were performed for each synaptic protein separately as continuous variable including age at diagnosis, sex, site of onset, and diagnostic delay as covariates. This revealed negative contributions to survival of high CSF levels of SNAP25, yet similar observations were seen for CSF levels of NRGN and SNCB (Supplementary Table 3). High levels of VAMP2 were borderline not significant (*P* = 0.053). However, upon adding CSF NfL levels to the multivariate analysis, significance of all synaptic proteins was lost (Supplementary Table 4).

### Synaptic proteins reflect different mechanisms as known markers of ALS pathology

**Figure 4:**
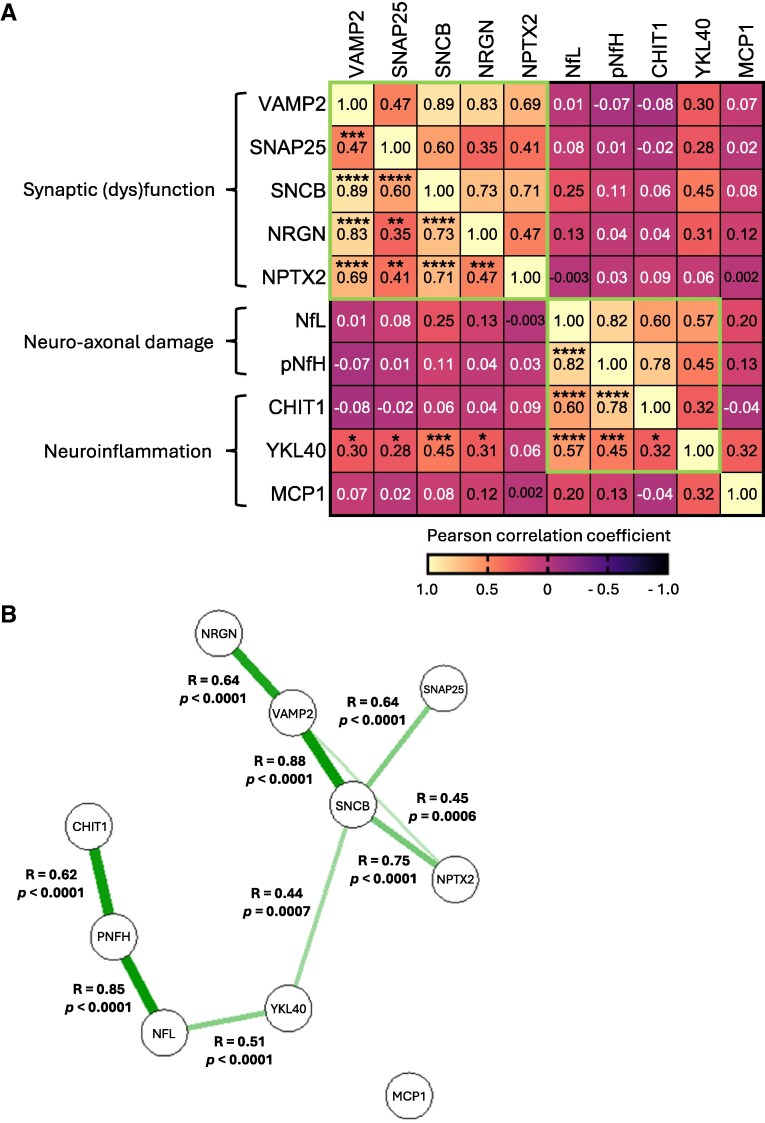
**Correlations of synaptic proteins with established ALS biomarkers.** (**A**) Pearson correlation matrix demonstrating the interplay of synaptic proteins with known ALS biomarkers (*P < 0.05; **P < 0.01; ***P < 0.001; ****P < 0.0001). *N* = 55. (**B**) Graphical lasso confirms a link between synaptic dysfunction and neuroinflammation. R = Pearson correlation coefficient. Bonferroni correction *P* = 0.005. *N* = 55. ALS, Amyotrophic lateral sclerosis.

To explore the value of synaptic markers for ALS, we correlated the levels with previously measured markers of neuroinflammation (MCP-1, YKL-40, CHIT-1) and neurofilaments (NfL, pNfH). Subsequently, we examined if synaptic pathology is related to neuro-axonal damage and neuroinflammation in ALS. To investigate this, we produced a correlation matrix including CSF biomarkers of neuroinflammation, neurofilaments and synaptic dysfunction (Figure 4A). These correlations were further studied in an elastic net regression analysis (Supplementary Fig. 5).

We confirmed the strong correlation between NfL and pNfH. In addition, strong correlations were observed between some of the synaptic proteins (Fig. 4B). We observed similar inter-synaptic correlations in controls and ALS patients, within pre-synaptic proteins as well as between pre-and post-synaptic proteins (Supplementary Fig. 6). No differences were observed in correlations between ALS patients and controls.

Interestingly, markers of synaptic dysfunction did not correlate with neurofilament levels suggesting that they may be surrogate biomarkers that display different pathological mechanisms in ALS. (Figure 4B) YKL-40 seemed to take a central position in ALS pathology, as it correlated weakly with both synaptic markers and neurofilaments.

## Discussion

There is an unmet need for ALS biomarkers as objective readout for different pathophysiological mechanisms underlying disease progression. The lack of such objective readouts and endpoints for clinical trials hampers treatment development efforts. Neurofilaments are well-established markers of axonal injury and have value as diagnostic and prognostic biomarker in ALS, since they are elevated in ALS and predict clinical progression and survival. As neurofilament levels are relatively stable throughout the disease, they can be used as marker for treatment response. However, they do not capture the full spectrum of underlying disease mechanisms and remain relatively stable throughout the disease trajectory. In this study, we explored the potential of synaptic markers in ALS, as synaptopathy has been implicated as an early event in ALS.^4,5^

We observed increased CSF levels of NRGN and VAMP2 in ALS patients, whereas no differences were observed for CSF NPTX2, SNCB and SNAP25. We observed high overlap between ALS patients and controls, indicating that synaptic proteins do not have diagnostic potential. Moreover, since we did not add ALS mimics or other neurodegenerative diseases in this study, the specificity of the synaptic biomarkers to reflect ALS pathology rather than general neurodegeneration remains to be investigated. Levels of SNAP25, SNCB and VAMP2 correlated cross-sectionally with relative disease stage, similar to NfL, pNfH, MCP-1 and YKL-40. Our findings warrant further validation in larger studies, including longitudinal samples and samples from presymptomatic individuals.

High levels of SNAP25 were consistently associated with a shorter survival time, indicating that CSF SNAP25 has prognostic value. However, the contributions of synaptic proteins to survival were lost upon adding NfL. We did not detect correlations between CSF synaptic proteins and neurofilaments, confirming that synaptic proteins in CSF do not merely reflect synaptic breakdown upon axonal damage. This could either be explained by different pathological mechanisms reflected by synaptic proteins in CSF or the temporal pattern of synaptic dysfunction compared with axonal damage. Whether these increased levels of synaptic markers precede neurofilament elevations cannot be answered by the current study and again emphasizes the importance of longitudinal studies.

Based on our correlation analyses, we hypothesized an interplay between synaptic dysfunction, neuroinflammation and axonal damage. YKL-40 is a pro-inflammatory protein secreted by astrocytes and YKL-40 levels are increased in CSF of ALS patients, where high levels are associated with shorter survival.^21^ Since YKL-40 correlates with both SNCB and NfL, we hypothesize that astrocytic hyper reactivity may be involved in synaptic dysfunction and axonal damage. The link between astrocytic hyperactivity and synaptic dysfunction is a well-established interplay contributing to neuronal degeneration and might be mediated by factors that activate astrocytes to prune synapses such as Megf10, Mertk, Ephrins and/or SPARC.^28^

We observed increased CSF levels of NRGN. As fluid biomarker, increased levels of NRGN have been investigated widely in AD.^29-31^ As speculated in AD, reduced tissue levels of NRGN and increased CSF levels of NRGN might indicate dendritic release of NRGN following synaptic injury and neuronal death.^29^ Earlier proteomics studies reported decreased levels of NRGN in synaptosomes of the frontal cortex of ALS patients.^32^ Interestingly, NRGN levels are actively upregulated in response to NO-induced oxidative stress, which induces synaptic damage.^33,34^ Therefore, the increased levels in CSF might either reflect dendritic injury or a compensatory upregulation to mediate excessive oxidative stress contributing to ALS pathology.

Moreover, we observed increased CSF levels of VAMP2 in ALS patients. Despite its functional relevance, VAMP2 has not yet been studied in biofluids of ALS patients.^35,36^ Interestingly, rare ALS-associated variants in *KIF1A* showed colocalization of KIF1A with VAMP2 on synaptic vesicle precursors.^37^ Whereas induced ubiquitination of VAMP2 leads to impaired synaptic transmission and degeneration of presynaptic motor nerve terminals, neuronal expression of human *G85R SOD1* in *C. elegans* results in decreased synaptobrevin intensity, which demonstrates vesicle recycling defects.^38,39^ Therefore, dysfunctional VAMP2 might contribute to ALS pathology. Because of its presence on the vesicular membrane, increased CSF levels of VAMP2 might indicate increased exosomal or (pre-)vesicular release. This is in line with recent findings demonstrating that synaptic proteins such as SNAP25 are present in extracellular vesicles in AD patients.^40^ Yet, if other synaptic proteins besides SNAP25 are present in exosomes and whether their role is neuroprotective or neurotoxic remains to be investigated.

Recently, NPTX2 was proposed to be an important dysregulated synaptic target of TDP-43 mediated loss-of-function in ALS.^15^ In addition, we recently demonstrated that high serum NPTX2 negatively contributes to survival.^41^ However, we did not find CSF NTPX2 levels to be altered or predictive of survival. Due to the expression of NPTX2 by endocrine organs, it has to be noted that the origin of NPTX2 in CSF and serum differs, which might underlie different contributions to survival. We observed a relative high variability of NPTX2 levels in CSF of controls, which complicated further analyses. Additionally, our results are contradictive to a recent proteomics study, demonstrating decreased NPTX2 levels in CSF, which might be due to methodological differences, since the epitopes detected in the NPTX2 immunoassay differ from the sequences used for the proteomic quantification by Oh *et al*.^42^ Recent research showed that reduced levels of NPTX2 in CSF of AD patients are a predictor of cognitive decline and NPTX2 can distinguish between several cognitive impairment disorders, including bvFTD.^43,44^ Additionally, *NPTX2*-deficient mice display increased complement activity, thereby extensively pruning synapses, which is in line with detrimental glial hyperactivity in ALS. The same study demonstrated decreased levels of NPTX2 with increased CSF levels of C1q.^45^ Since NPTX2 is involved in formation of excitatory synapses, the differential susceptibility of excitatory and inhibitory synapses of both cortical and spinal origin might underlie the lack of significant differences of CSF NPTX2 levels in ALS.^9^

Remarkably, although CSF levels of SNCB and SNAP25 were not different between ALS and controls, high levels of SNAP25 (and to lesser extent SNCB) contribute to survival. This might indicate that these presynaptic proteins might be released in CSF upon ALS pathology, but extensive presynaptic loss in ALS patients might mask any possible differences compared with controls. Therefore, a possible increase in CSF levels contributing to worsened survival might be undetectable.

Increased levels of SNCB have been mainly associated with AD and Lewy body dementia.^46,47^ Additionally, decreased SNCB levels have been demonstrated in synaptosomes of *C9orf72* patients.^32^ Previous evidence of ALS as a synucleinopathy can link synaptic defects with synuclein alterations in ALS, yet this is preliminary and should be investigated further.^48^ The robust link between SNCB and YKL-40 might be explained by the fact that SNCB inhibits phospholipase D2, which is secreted by astrocytes and mediates cellular inflammation.^49^ Importantly, inhibition of phospholipase D2 has been shown to ameliorate ALS phenotypes in *SOD1* mice and *Drosophila*.^50,51^ Therefore, neuroinflammation due to glial hyperactivity might result in SNCB alterations that contribute to worsened survival.

Despite its association with survival, the lack of a difference in CSF SNAP25 levels between patients and controls is striking. While SNAP25 is increased in AD CSF, SNAP25 is also detected in serum neuronal-derived exosomes of AD and stroke patients.^40,52^ In mutant *SOD1* ALS patients, suppression of SNAP25 activity is shown, whereas no altered levels of SNAP25 are shown in anterior horn cells of sporadic ALS patients.^53,54^ In skeletal muscle of mutant *SOD1* mice, SNAP25 levels were decreased at the presymptomatic stage, but recovered at later stages, indicating dynamic alterations in SNAP25 expression.^55^ In mutant *FUS* mice, SNAP25 demonstrated presynaptic enrichment upon FUS accumulation in synaptic boutons.^56^ Therefore, similar to SNCB, the release, expression or activity of SNAP25 might be altered following ALS pathology, but undetectable in CSF due to temporal dynamics or synaptic loss.

In AD, several other synaptic markers have been studied in both serum and CSF, and the elevations in early disease stages reflect presymptomatic synaptic dysfunction or loss. Recent research in CSF of AD patients demonstrated increased levels of SNAP25, VAMP2 and NRGN, with some correlating with cognitive decline and AD pathology.^30,57,58^ More specifically, CSF levels of SNAP25, NRGN and VAMP2 have prognostic value in AD, as decreased levels are observed in stage 1 preclinical AD, but increased levels are observed in later disease stages.^57^ This has not yet been investigated in ALS and should be performed in large longitudinal cohorts, including presymptomatic measurements. Intriguingly, CSF levels of SNAP25 and NRGN are able to distinguish AD from behavioural frontotemporal dementia (bvFTD), indicating pathology-dependent differences.^31^

The observed similarities and differences between synaptic proteins in CSF of AD and ALS patients indicates that, although synaptic dysfunction is present in both pathologies, the extent of synaptic dysfunction contributing to disease pathology might differ. One obvious explanation underlying differences is the number of neurons affected by ALS and AD. Whereas the whole cortex is affected in AD, ALS pathology is mostly restricted to neurons in motor and some frontal regions. Secondly, ALS is far more aggressive than AD, thus differences could also be influenced by the rapid loss of neurons in ALS compared with AD. Lastly, the average age of onset in AD is higher compared with ALS, and possible contributions of ageing on CSF biomarker levels must be taken into account.

Altogether, we show that NRGN and VAMP2 are increased in CSF of ALS patients. Moreover, several synaptic proteins (including proteins that were not increased in CSF) correlated with relative ALS disease stage and SNAP25 was predictive of the overall survival, indicating the dynamic nature of synaptic proteins as biomarkers. It needs to be determined if synaptic markers change over time and if change is related to survival. We hypothesize that increased synaptic proteins display either synaptic loss or increased release of exosomes or pre-synaptic vesicles upon neuronal/synaptic damage, which might arise at an earlier timepoint than axonal breakdown and neuroinflammation and might contribute to synaptic dysfunction and ALS pathology.

## Supplementary Material

fcag247_Supplementary_Data

## Data Availability

Raw data for biomarker levels and clinical parameters can be shared upon reasonable request.
