# Supplementary material for: Value of synaptic proteins as biomarkers in amyotrophic lateral sclerosis
Source: Brain Commun. 2026 Jun 25;8(4):fcag247. doi: 10.1093/braincomms/fcag247 (PMC13332409; doi:10.1093/braincomms/fcag247)
Supplement: fcag247_Supplementary_Data [file fcag247_supplementary_data.zip › Supplementary_material.docx]

**Supplementary material**

|  | Age | Disease duration | Progression rate  at diagnosis | ALS-FRS-R total score | ALS-FRS-R slope | First weight measured | Weight loss  at diagnosis (%) | Summed CMAP (mV) |
| --- | --- | --- | --- | --- | --- | --- | --- | --- |
| NRGN | 0.15 | -0.013 | 0.19 | -0.065 | 0.28 | -0.059 | -0.099 | 0.0066 |
| VAMP2 | 0.065 | 0.17 | 0.13 | -0.11 | 0.18 | 0.050 | -0.32 | -0.065 |
| SNAP25 | 0.10 | -0.095 | 0.16 | -0.18 | 0.24 | 0.076 | -0.21 | 0.061 |
| NPTX2 | -0.11 | 0.12 | 0.025 | 0.029 | 0.062 | 0.23 | -0.26 | 0.021 |
| SNCB | 0.20 | 0.10 | 0.15 | -0.14 | 0.21 | -0.11 | -0.28 | -0.099 |

**Supplementary Table 1.** **Correlations of synaptic proteins in CSF with clinical parameters.** Correlation values are indicated as Spearman or Pearson R coefficients. The values of CSF ALS and CSF CTRL are the concentrations of each protein in pg/mL. ALS-FRS-R = Revised amyotrophic lateral sclerosis functional rating scale. CMAP = compound muscle action potential.

|  | Age | First weight measured |
| --- | --- | --- |
| NRGN | 0.13 | 0.074 |
| VAMP2 | 0.11 | -0.035 |
| SNAP25 | 0.33 | 0.14 |
| NPTX2 | 0.11 | 0.23 |
| SNCB | 0?17 | -0.038 |

**Supplementary. Table. 2.** Correlations of synaptic proteins in CSF with clinical parameters in controls. Values are given as Pearson or Spearman R, depending on the normality of the data.

|  | Cox *p*-value | Hazard ratio [95% CI] |
| --- | --- | --- |
| Age | 0.482 | 1.012 [0.978 – 1.047] |
| Male | 0.977 | 0.990 [0.513 – 1.912] |
| Site of onset (spinal) | 0.197 | 0.585 [0.259 – 1.320] |
| Diagnostic delay | 0.822 | 0.995 [0.951 – 1.041] |
| SNAP25 | 0.025 | 1.401 [1.043 – 1.882] |
| Age | 0.394 | 1.015 [0.981 – 1.050] |
| Male | 0.575 | 1.218 [0.612 – 2.421] |
| Site of onset (spinal) | 0.164 | 0.559 [0.246 – 1.267] |
| Diagnostic delay | 0.520 | 0.982 [0.930 – 1.037] |
| SNCB | 0.013 | 1.761 [1.125 – 2.758] |
| Age | 0.486 | 1.012 [0.978 – 1.047] |
| Male | 0.950 | 0.979 [0.507 – 1.890] |
| Site of onset (spinal) | 0.305 | 0.651 [0.287 – 1.479] |
| Diagnostic delay | 0.577 | 0.985 [0.936 – 1.038] |
| NRGN | 0.002 | 1.841 [1.241 – 2.732] |
| Age | 0.451 | 1.013 [0.979 – 1.048] |
| Male | 0.553 | 1.229 [0.622 – 2.428] |
| Site of onset (spinal) | 0.159 | 0.549 [0.239 – 1.264] |
| Diagnostic delay | 0.619 | 0.988 [0.941 – 1.037] |
| VAMP2 | 0.028 | 1.585 [1.050 – 2.392] |
| Age | 0.498 | 1.014 [0.974 – 1.056] |
| Male | 0.925 | 1.036 [0.500 – 2.144] |
| Site of onset (spinal) | 0.299 | 0.654 [0.294 – 1.457] |
| Diagnostic delay | 0.828 | 0.995 [0.952 – 1.040] |
| NPTX2 | 0.775 | 1.070 [0.672 – 1.7060] |

**Supplementary Table 3. Multivariate cox regression model reveals relative contributions of synaptic proteins to survival.** Values are displayed as Cox *p*-values and hazard ratios (HR) with 95% confidence intervals (CI). Normalized CSF levels of synaptic proteins are included as continuous variables. Bonferroni correction: *p* = 0.01.

|  | Cox *p*-value | Hazard ratio [95% CI] |
| --- | --- | --- |
| Age | 0.48 | 1.036 [1.000 – 1.072] |
| Male | 0.757 | 0.899 [0.458 – 1.765] |
| Site of onset (spinal) | 0.065 | 0.459 [0.200 – 1.050] |
| Diagnostic delay | 0.930 | 0.998 [0.947 – 1.051] |
| NfL | <0.001 | 3.544 [2.089 – 6.014] |
| SNAP25 | 0.062 | 1.361 [0.984 – 1.883] |
| Age | 0.068 | 1.032 [0.998 – 1.067] |
| Male | 0.757 | 0.895 [0.442 – 1.810] |
| Site of onset (spinal) | 0.062 | 0.451 [0.196 – 1.041] |
| Diagnostic delay | 0.992 | 1.000 [0.942 – 1.061] |
| NfL | <0.001 | 3.339 [1.865 – 5.977] |
| SNCB | 0.715 | 1.098 [0.663 – 1.819] |
| Age | 0.058 | 1.033 [0.999 – 1.069] |
| Male | 0.633 | 0.848 [0.430 – 1.670] |
| Site of onset (spinal) | 0.078 | 0.467 [0.200 – 1.088] |
| Diagnostic delay | 0.843 | 0.994 [0.941 – 1.051] |
| NfL | <0.001 | 3.272 [1.869 – 5.728] |
| NRGN | 0.232 | 1.320 [0.838 – 2.080] |
| Age | 0.072 | 1.032 [0.997 – 1.067] |
| Male | 0.881 | 0.949 [0.474 – 1.898] |
| Site of onset (spinal) | 0.042 | 0.412 [0.175 – 0.970] |
| Diagnostic delay | 0.998 | 1.000 [0.946 – 1.057] |
| NfL | <0.001 | 3.659 [2.082 – 6.432] |
| VAMP2 | 0.603 | 1.129 [0.715 – 1.781] |
| Age | 0.183 | 1.027 [0.988 – 1.068] |
| Male | 0.632 | 0.839 [0.410 – 1.718] |
| Site of onset (spinal) | 0.054 | 0.436 [0.187 – 1.015] |
| Diagnostic delay | 0.916 | 1.003 [0.953 – 1.055] |
| NfL | <0.001 | 3.668 [2.160 – 6.231] |
| NPTX2 | 0.672 | 0.903 [0.564 – 1.447] |

**Supplementary Table 4.** Cox regression analyses of clinical parameters with CSF levels of NfL, NRGN, SNCB, VAMP2 and NPTX2 in combination with CSF NfL. Each protein is normalized and analysed as continuous variable. Values are displayed as Cox *p*-values and hazard ratios (HR) with 95% confidence intervals (CI). Bonferroni correction: *p* = 0.01.

**
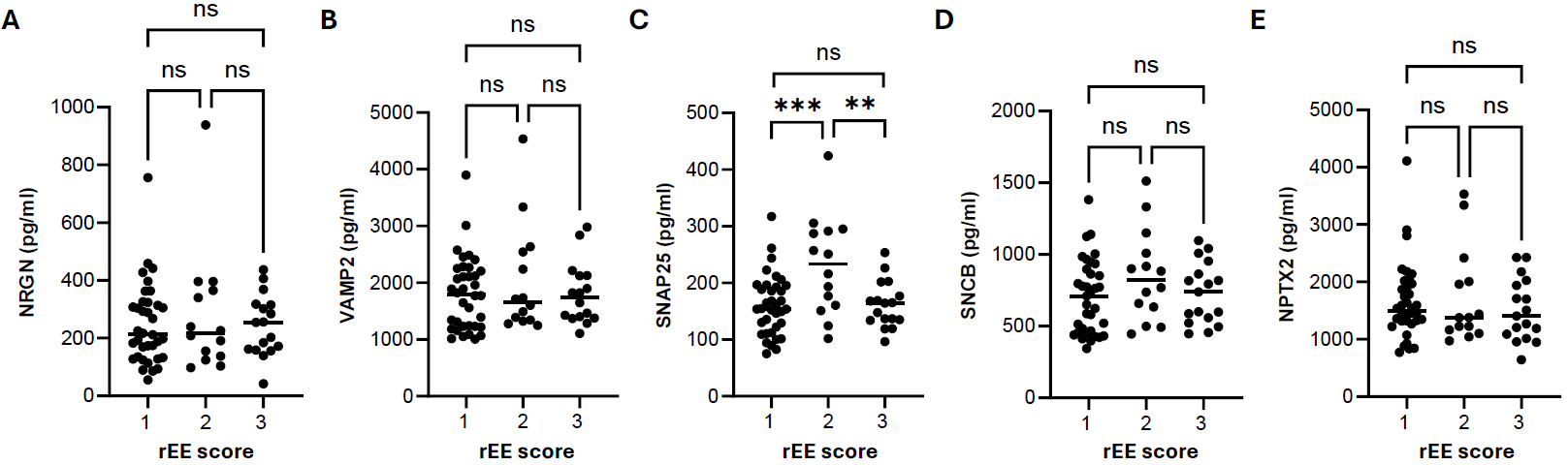
Supplementary Fig. 1.** CSF levels of synaptic biomarkers do not clearly correspond to the number of Revised El Escorial regions affected. **A.** NRGN: Kruskal-Wallis *p* = 0.96. N rEE 1 = 36; N rEE 2 = 14; N rEE 3 = 17. **B.** VAMP2: Kruskal-Wallis *p* = 0.79. N rEE 1 = 36; N rEE 2 = 14; N rEE 3 = 17. **C.** SNAP25: ANOVA *p* = 0.0009. Tukey’s multiple comparison 1 vs 2: *p* = 0.0010; 1 vs 3: *p* = 0.99; 2 vs 3: *p* = 0.0054. N rEE 1 = 36; N rEE 2 = 14; N rEE 3 = 17. **D.** SNCB: Kruskal-Wallis *p* = 0.26. N rEE 1 = 35; N rEE 2 = 14; N rEE 3 = 17. **E.** NPTX2: Kruskal-Wallis *p* = 0.78. N rEE 1 = 36; N rEE 2 = 14; N rEE 3 = 17. Data points represent biomarker levels from individual ALS patients. rEE = Revised El Escorial. NS = not significant.

**
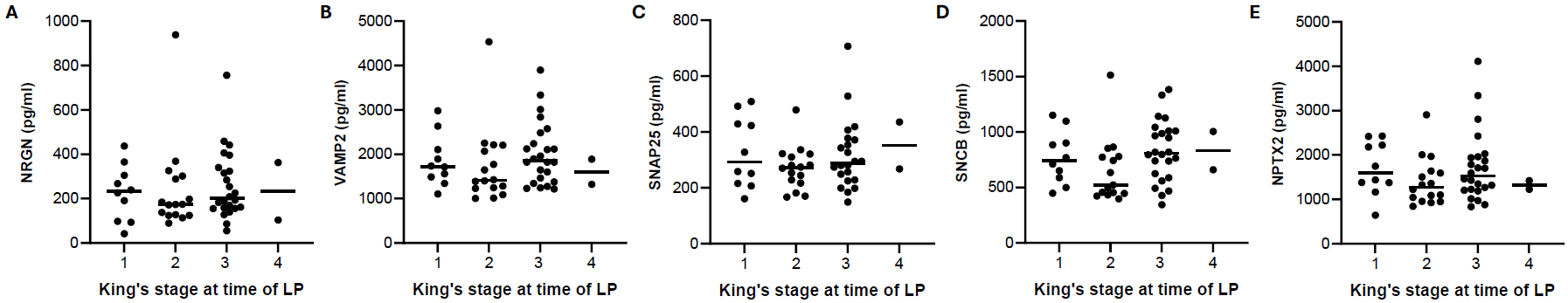

Supplementary Fig. 2.** CSF levels of synaptic biomarkers do not differ between groups depending on King’s stage at the time of the lumbar puncture (LP), **A.** NRGN: Kruskal-Wallis *p* = 0.82. N King’s 1 = 10; N King’s 2 = 16; N King’s 3 = 24; N King’s 4 = 2. **B.** VAMP2: Kruskal-Wallis *p* = 0.37. N King’s 1 = 10; N King’s 2 = 16; N King’s 3 = 24; N King’s 4 = 2. **C.** SNAP25: Kruskal-Wallis *p* = 0.57. N King’s 1 = 10; N King’s 2 = 16; N King’s 3 = 22; N King’s 4 = 2. **D.** SNCB: Kruskal-Wallis *p* = 0.14. N King’s 1 = 10; N King’s 2 = 15; N King’s 3 = 24; N King’s 4 = 2. **E.** NPTX2: Kruskal-Wallis *p* = 0.34. N King’s 1 = 10; N King’s 2 = 16; N King’s 3 = 24; N King’s 4 = 2. Data points represent biomarker levels from individual ALS patients.

**
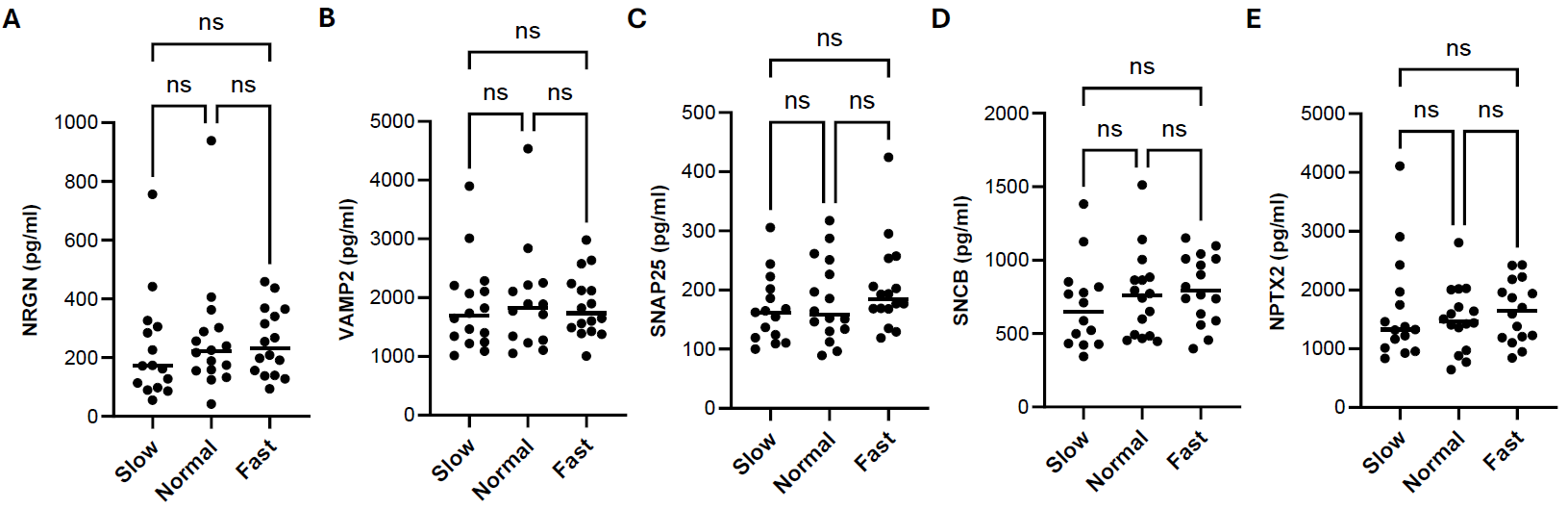
Supplementary Fig. 3.** CSF levels of synaptic biomarkers do not differ between groups depending on progression type. **A.** NRGN: Kruskal-Wallis *p* = 0.08. N Slow = 15; N Normal = 16. N Fast = 16. **B.** VAMP2: Kruskal-Wallis *p* = 0.17. N Slow = 16; N Normal = 14. N Fast = 16. **C.** SNAP25: Kruskal-Wallis *p* = 0.24. N Slow = 15; N Normal = 16. N Fast = 16. **D.** SCNB: Kruskal-Wallis *p* = 0.17. N Slow = 14; N Normal = 16. N Fast = 16. **E.** NPTX2: Kruskal-Wallis *p* = 0.62. N Slow = 15; N Normal = 16. N Fast = 16. Data points represent biomarker levels from individual ALS patients. NS = not significant.


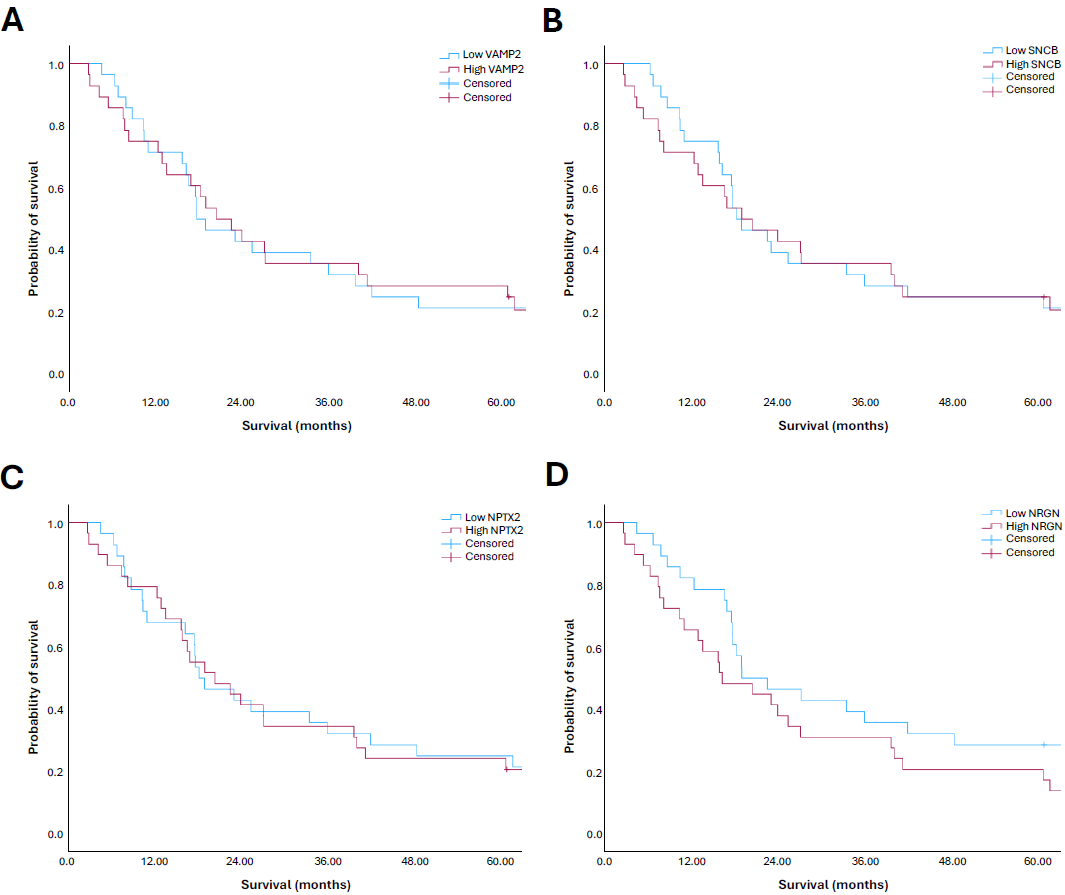


**Supplementary Fig. 4.** Kaplan-Meier analyses of low and high CSF levels of synaptic proteins. **A.** VAMP2: *p*-value = 0.98. **B.** SNCB: *p*-value = 0.82. **C.** NPTX2: *p*-value = 0.84. **D.** NRGN: *p*-value = 0.11. N = 57 for NRGN, NPTX2. N = 56 for SNCB and VAMP2.


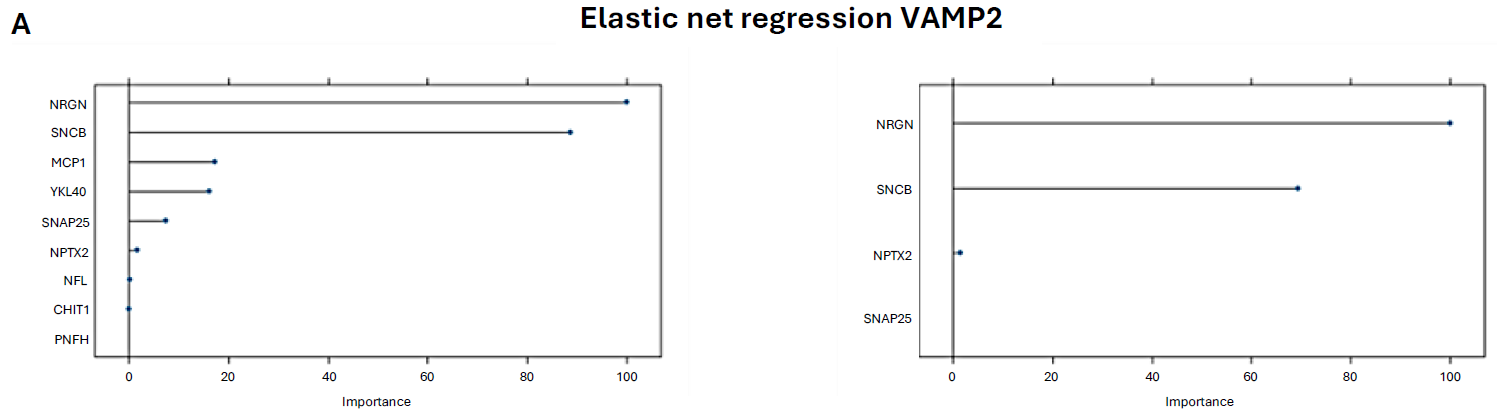


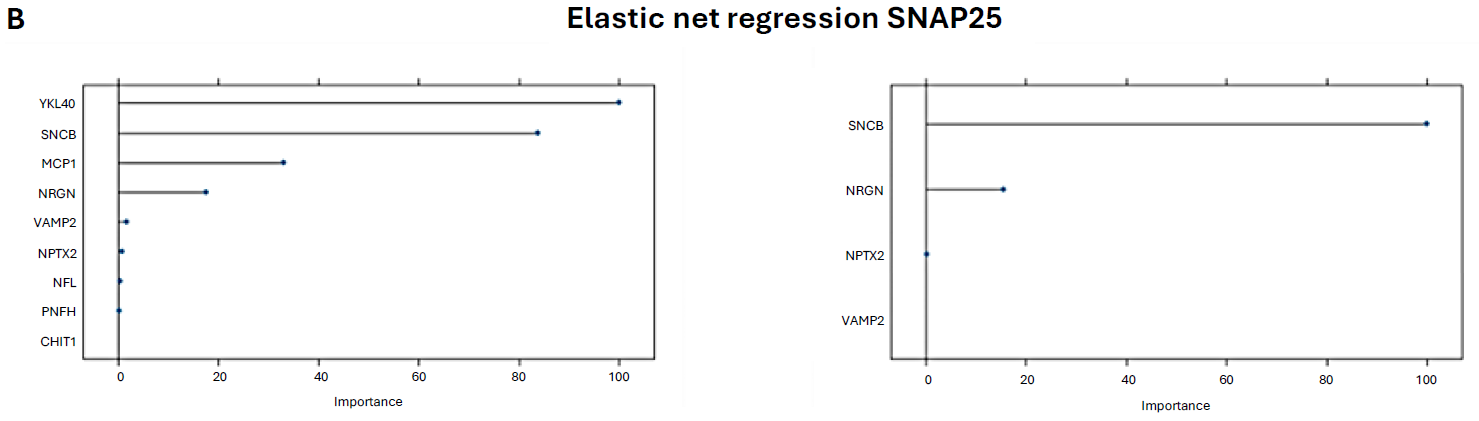


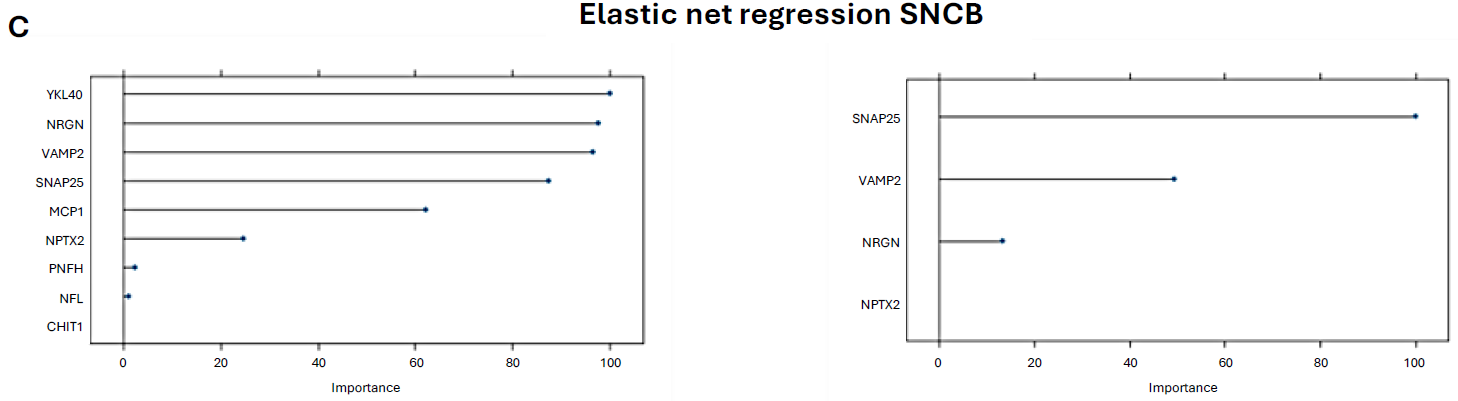


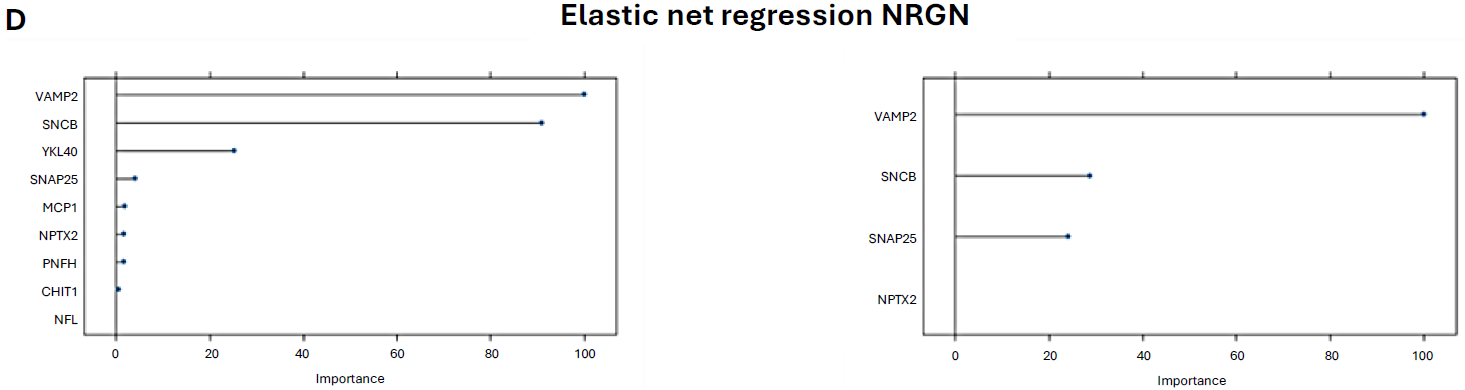


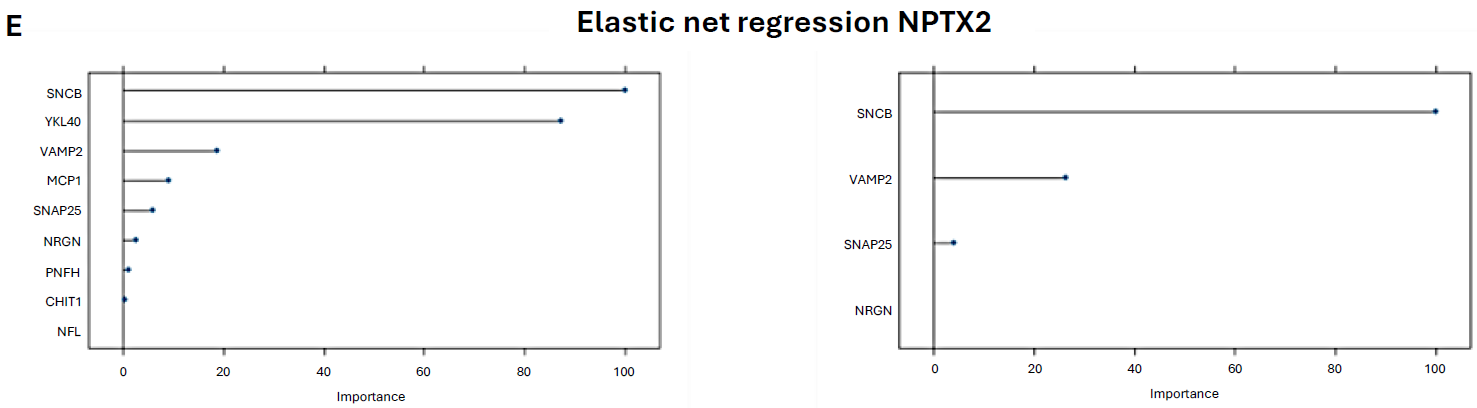


**Supplementary Fig. 5.** Elastic net regression analyses display interactions between synaptic proteins and established ALS biomarkers (left) and synaptic proteins only (right). **A.** Predicted correlations of VAMP2. **B.** Predicted correlations of SNAP25. **C.** Predicted correlations of SNCB. **D.** Predicted correlations of NRGN. **E.** Predicted correlations of NPTX2. N = 55 for all analyses.


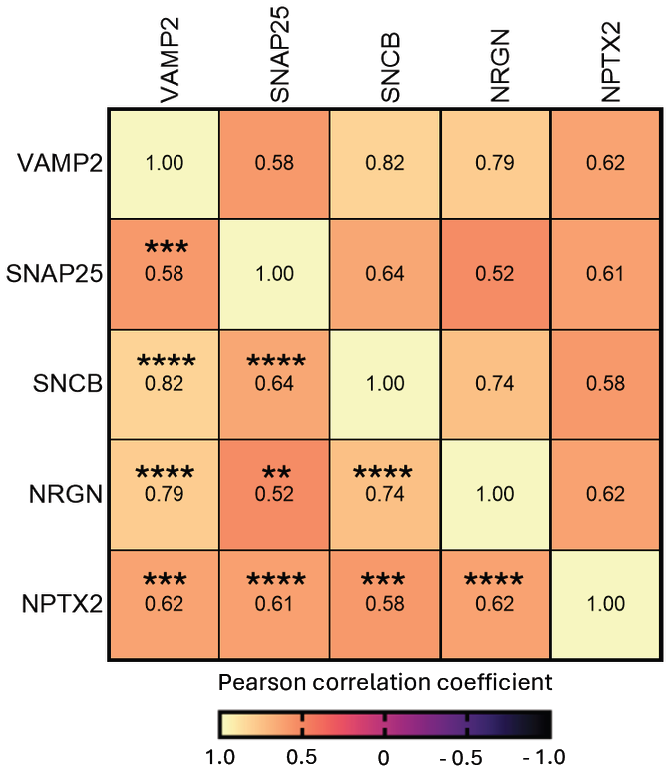


**Supplementary Fig. 6.** Pearson correlation matrix of synaptic proteins in controls. N = 36.
